# Plasma tRF-1:29-Pro-AGG-1-M6 and tRF-55:76-Tyr-GTA-1-M2 as novel diagnostic biomarkers for lung adenocarcinoma

**DOI:** 10.3389/fonc.2022.991451

**Published:** 2022-09-20

**Authors:** Jianbin You, Guoliu Yang, Yi Wu, Xuan Lu, Shuyu Huang, Qianshun Chen, Chen Huang, Falin Chen, Xunyu Xu, Liangyuan Chen

**Affiliations:** ^1^ Department of Clinical Laboratory, Shengli Clinical Medical College of Fujian Medical University, Fuzhou, China; ^2^ Department of Basic Medical Science, Xiamen Medical College, Xiamen, China; ^3^ Department of Clinical Laboratory, Zhangzhou Skin Disease Prevention and Treatment Hospital of Fujian Province, Zhangzhou, China; ^4^ Department of Blood Transfusion, The First Affiliated Hospital of Fujian Medical University, Fuzhou, China; ^5^ Department of Thoracic Surgery, Shengli Clinical Medical College of Fujian Medical University, Fuzhou, China

**Keywords:** tRFs/tiRNAs, lung adenocarcinoma, plasma, high-throughput sequencing, biomarkers

## Abstract

**Objective:**

TRNA-derived fragments (tRFs) and tRNA-derived stress-induced RNAs (tiRNAs) are recognized as novel and potential types of non-coding RNAs (ncRNAs), and several tRF/tiRNA signatures are closely associated with tumor diagnosis. This study aimed to analyze the expression profiles of plasma tRFs/tiRNAs and to clarify their diagnostic value in lung adenocarcinoma (LUAD).

**Methods:**

The differential expression profiles of plasma tRFs/tiRNAs in patients with four patients with early LUAD, four patients with advanced LUAD, and four healthy controls were analyzed using high-throughput sequencing technology. Then, plasma tRFs/tiRNAs were validated by quantitative real-time polymerase chain reaction (qRT-PCR), and their diagnostic efficiency was appraised by receiver operating characteristic curve analysis. The correlation of candidate plasma tRFs/tiRNAs with clinicopathological features was also analyzed. Finally, bioinformatics analysis was performed to explore and identify the potential biological pathways induced by tRFs/tiRNAs.

**Results:**

The sequencing results revealed that tRFs/tiRNAs from plasma samples in patients with LUAD were differently expressed, supporting the necessity of exploring their potential as biomarkers. The validation results of qRT-PCR demonstrated that the expression level of tRF-1:29-Pro-AGG-1-M6 was downregulated in LUAD, while that of tRF-55:76-Tyr-GTA-1-M2 was upregulated, which was consistent with the sequencing data. The areas under the receiver operating characteristic curve of tRF-1:29-Pro-AGG-1-M6 and tRF-55:76-Tyr-GTA-1-M2 were 0.882 and 0.896, respectively, which have significant values in the diagnosis of LUAD. The expressions of tRF-1:29-Pro-AGG-1-M6 and tRF-55:76-Tyr-GTA-1-M2 in LUAD were obviously correlated with various clinicopathological features such as tumor–node–metastasis stage, node stage, and the expression levels of carcinoembryonic antigen. In addition, their expression was significantly altered from before to after tumor resection in LUAD patients. The results of Gene Ontology and Kyoto Encyclopedia of Genes and Genomes analyses further indicated that tRF-1:29-Pro-AGG-1-M6 and tRF-55:76-Tyr-GTA-1-M2 are widely distributed and apparently enriched in several tumor-related signaling pathways.

**Conclusions:**

Plasma tRF-1:29-Pro-AGG-1-M6 and tRF-55:76-Tyr-GTA-1-M2 may be promising components in the development of highly sensitive and non-invasive biomarkers for LUAD diagnosis.

## Introduction

One of the malignant tumors that most significantly threaten human life worldwide is lung cancer, and the subtype with the highest incidence is lung adenocarcinoma (LUAD) (1, 2). Despite considerable progress made in treatment, the 5-year overall survival rate for LUAD is ≤15%, as a large proportion of patients are often diagnosed at a later stage of the disease when symptoms become apparent (3). Although low-dose computed tomography is the early diagnostic method with the highest rate of LUAD detection, its typical defects of a high price and false-positive rate are apparent (4). Additionally, traditional serum biomarkers such as carcinoembryonic antigen (CEA) and cytokeratin fragment antigen 21-1 often fail to achieve early diagnosis of LUAD due to insufficient specificity (5). The discovery of new early diagnostic biomarkers is of great importance to overcome LUAD, which will provide practical and effective support for the timely detection, early treatment, and even improved prognosis of the condition.

With the assistance of high-throughput sequencing technology, a large quantity of non-coding RNAs have been identified to play important roles in the occurrence and progression of cancer (6). Transfer RNA (tRNA)-derived small non-coding RNAs (tsRNAs) have received extensive attention, and the group’s main members, tRNA-derived fragments (tRFs) and tRNA-derived stress-induced RNAs (tiRNAs), are obtained from precursor or mature tRNAs through specific cleavage by nucleases (7, 8). The understanding of tRFs/tiRNAs can be traced back to the 1970s when Borek et al. (9) found that an abundant supply of tRFs/tiRNAs in tumor tissue originated from a high turnover of tRNAs. Since then, successive studies have detected large amounts of tRFs/tiRNAs in cells, tissues, and body fluids (10–12) and have demonstrated that the stable existence of tRFs/tiRNAs has crucial regulatory functions in gene expression, protein translation, and epigenetic modification (13–15). The latest evidence reveals that tRFs/tiRNAs are abnormally expressed in neurological diseases, metabolic diseases, and cancer (16). Specifically, research has determined that tiRNA-Gln-CTG-003, tiRNA-His-GTG-001, and tRF-Ala-AGC-002 are abnormally expressed in advanced ovarian cancer tissues, while 5′-tRF-LysCTT appears to be overexpressed in bladder cancer patients (17, 18). Moreover, Shao et al. (19) indicated that the level of tRF-Leu-CAG was upregulated in non-small cell lung cancer (NSCLC) tumor tissues and cell lines and observed that its expression in NSCLC serum significantly correlated with tumor stage progression. Li et al. (20) discovered that serum tRF-31-79MP9P9NH57SD expression was higher in NSCLC patients and related to both clinical stage and lymph node malignancy. It is reasonable to believe that tRFs/tiRNAs can serve as candidate molecular markers for monitoring cancer.

In the present study, based on high-throughput sequencing technology, the plasma tRF/tiRNA expression profiles of four patients with early LUAD, four patients with advanced LUAD, and four healthy controls were analyzed. First, tiRNA-1:34-Val-CAC-2, tRF-1:29-Pro-AGG-1-M6, and tRF-55:76-Tyr-GTA-1-M2 were screened out as candidate tRFs/tiRNAs and verified by quantitative real-time polymerase chain reaction (qRT-PCR). Then, the diagnostic efficacy of plasma tRFs/tiRNAs was assessed by receiver operating characteristic (ROC) curve analysis. Subsequently, this study predicted the potential target genes of tRFs/tiRNAs and their regulatory networks using bioinformatics technology then further explored their main cellular biological functions and related molecular mechanisms in LUAD.

## Materials and methods

### Clinical information

All plasma samples were collected from LUAD patients and healthy individuals who visited Fujian Provincial Hospital between January 2021 and March 2022.

A total of 19 men and 28 women aged 28–80 years with an average age of 56.02 ± 10.42 years and histopathologically confirmed LUAD were enrolled. We excluded any patients with hypertension, diabetes, severe liver and kidney disease, metastatic tumors, or other systemic diseases. The stage of LUAD was defined according to the version of the tumor–node–metastasis (TNM) system released by the Union for International Cancer Control/American Joint Committee on Cancer. Separately, the healthy control group included 9 men and 12 women aged 26–77 years with an average age of 47.71 ± 12.48 years without lung disease, tumors, or various systemic diseases.

Plasma samples from four patients with early LUAD, four patients with advanced LUAD, and four healthy controls were randomly selected for sequencing analysis, and the remaining samples were reserved for consecutive studies. In addition, plasma samples from 47 LUAD patients were selected for qRT-PCR analysis, and plasma samples from 12 LUAD patients were selected to evaluate the changes in tRF/tiRNA expression after complete tumor resection. Relevant clinical data were collected from all participants and recorded in detail. Written informed consent was given by all participants, and the study was approved by the ethics committee of Fujian Provincial Hospital (K2021-03-054).

### Extraction and pretreatment of plasma RNA

Total RNA was extracted from plasma using TRIzol™ LS reagent (Thermo Fisher Scientific, Waltham, MA, USA). The concentration and purity of each RNA sample were assessed with an ND-1000 spectrophotometer (Thermo Fisher Scientific). The absorbance at wavelengths of 260 and 280 nm was measured. All the isolated RNA samples had an OD260/OD280 ratio between 1.8 and 2.1. The RNA integrity was checked by agarose gel electrophoresis. Total RNA samples were pretreated to remove some RNA modifications that interfere with small RNA sequencing library construction according to the following process: 3′-aminoacyl (charged) was deacylated to 3′-OH for 3′-adaptor ligation, 3′-cP (2’,3′-cyclic phosphate) was removed to 3′-OH for 3′-adaptor ligation, 5′-OH (hydroxyl group) was phosphorylated to 5′-P for 5′-adaptor ligation, and m1A and m3C were demethylated for efficient reverse transcription.

### Library preparation and tRF/tiRNA sequencing

Pretreated total RNA was used to prepare the sequencing library. First, the total RNA of each sample was sequentially ligated to 3′ and 5′ small RNA adapters. Complementary DNA (cDNA) was then synthesized and amplified using proprietary RT and amplification primers (Illumina, San Diego, CA, USA). Subsequently, 134–160-bp PCR-amplified fragments were extracted and purified from the polyacrylamide gel electrophoresis gel. Finally, the completed libraries were quantified with the Agilent 2100 bioanalyzer (Agilent Technologies, Santa Clara, CA, USA). The libraries were denatured as single-stranded DNA molecules, captured on Illumina flow cells, amplified *in situ* as sequencing clusters, and sequenced for 50 cycles on the Illumina NextSeq 500 system per the manufacturer’s instructions.

### Sequencing data analysis of tRFs/tiRNAs

Image analysis and base calling were performed using Solexa pipeline version 1.8 (Off-Line Base Caller software, version 1.8). Sequencing quality was examined in FastQC (Babraham Institute, Cambridge, UK). Raw data files in FastQC format were generated from the Illumina sequencer. To examine the sequencing quality, the quality score plot of each sample was plotted. The quality score Q was logarithmically related to the base calling error probability (P). After Illumina quality control, the sequencing reads were 5,3-trimmed, and reads (length of <14 or >40 nt) with cutadapt were discarded and recorded in FASTA format. Trimmed reads in FASTA format were aligned allowing for one mismatch only to the mature tRNA sequences; then, reads that did not map were aligned, allowing for one mismatch only to precursor tRNA sequences with the bowtie software. Subsequently, the remaining reads were aligned, allowing for one mismatch only to microRNA reference sequences with miRDeep2. The abundance of tRFs/tiRNAs was evaluated using their sequencing counts and was normalized as counts per million of total aligned reads (CPM). Based on alignment statistical analysis (mapping ratio, read length, and fragment sequence bias), we determined whether the results could be used for subsequent data analysis. If so, the expression profiling and differentially expressed tRFs/tiRNAs and miRNAs were calculated. A fold change ≥1.5 and *p* ≤ 0.05 were used for screening differentially expressed tRFs/tiRNAs. Principal component analysis, correlation analysis, pie plots, Venn plots, hierarchical clustering, scatterplots, and volcano plots were performed for the expressed tRFs/tiRNAs in the R (R Foundation for Statistical Computing, Vienna, Austria) or perl environment for statistical computing and graphics.

### qRT-PCR analysis

Following the manufacturer’s instructions, RNA pretreatment and cDNA synthesis were performed using the rtStar™ tRF & tiRNA Pretreatment Kit and the rtStar™ First-Strand cDNA Synthesis Kit, respectively (both Arraystar, Rockville, MD, USA). According to the 2× PCR Master Mix Kit manufacturer’s protocol, synthetic cDNA was analyzed by qRT-PCR on a LightCycler480 real-time quantitative PCR system (Roche Holding, Basel, Switzerland). All reactions were conducted in triplicate, and the relative expression levels of tRFs/tiRNAs were computed using the 2^−ΔΔCt^ and 2^−ΔCt^ methods with U6 as an internal control. The primers of targeted genes were as follows: U6, forward 5′-GCTTCGGCAGCACATATACTAAAAT-3′ and reverse 5′-CGCTTCACGAATTTGCGTGTCAT-3′; tiRNA-1-34-Val-CAC-2, forward 5′-ACGATCGCTTCTGTAGTGTAGTGG-3′ and reverse 5′-CGATCTGAGGCGAACGTGAT-3′; tRF-55-76-Tyr-GTA-1-M2, forward 5′-CAGTCCGACGATCTCGAATCC-3′ and reverse 5′-GCTCTTCCGATCTTGGTCCTTC-3′; and tRF-1-29-Pro-AGG-1-M6, forward 5′-GATCGGCTCGTTGGTCTAGG-3′ and reverse 5′-CTTCCGATCTCGAGAATCATACC-3′.

### Determination of carcinoembryonic antigen, neuron-specific enolase, and squamous cell carcinoma (SCC) concentrations in serum by electrochemiluminescence

Following the manufacturer’s instructions, the expression levels of CEA, neuron-specific enolase (NSE), and SCC in serum were quantified on a Cobas E602 machine (Roche Holding) using the original kit. The cutoff values for CEA, NSE, and SCC were 5, 16.3, and 2.7 ng/ml, respectively.

### Bioinformatics analysis of tRFs/tiRNAs

The exact location of each tRF in the secondary structure of the derived tRNA was determined according to the GtRNAdb database (http://gtrnadb.ucsc.edu/). Then, potential target genes of tRFs/tiRNAs were explored in the TargetScan (http://www.targetscan.org/vert_72/) and Miranda (http://www.microrna.org/microrna/) databases. Gene Ontology (GO) and Kyoto Encyclopedia of Genes and Genomes (KEGG) functional enrichment of tRFs/tiRNAs was described using KOBAS version 3.0.

### Statistical analysis

Statistical analysis was carried out by using SPSS Statistics version 24.0 (IBM Corporation, Armonk, NY, USA) and GraphPad Prism version 8.0 software (GraphPad Software, La Jolla, CA, USA). An unpaired *t*-test was employed to assess the differences between all LUAD patients and healthy controls. In addition, an unpaired *t*-test was used to analyze the correlation between tRFs/tiRNAs and clinicopathological features, and a paired *t*-test was carried out to assess the expression differences between preoperative and postoperative LUAD patients. The area under the ROC curve (AUC) was calculated based on the ROC curve to determine the diagnostic value. All measurement data are presented as the mean and standard error of the mean (SEM). Youden’s index was used to calculate the optimal cutoff values of the tRFs/tiRNAs, and we considered *p* < 0.05 to be statistically significant.

## Results

### Expression profiles of tRFs/tiRNAs in plasma

**Figure 1:**
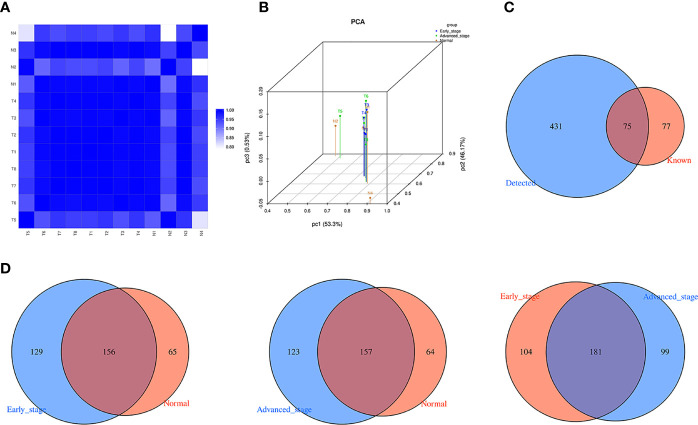
The expression profiles of tRFs/tiRNAs in plasma between LUAD patients and healthy controls. **(A)** Correlation coefficient heatmap for all samples, with colors in panels closer to blue representing higher correlation coefficients between the two samples. **(B)** PCA between LUAD patients and healthy controls based on tRF/tiRNA expression profiles. **(C)** Venn diagram shows the number of tRFs/tiRNAs detected in this project and collected in the tRFdb. **(D)** Venn diagram of commonly and specifically expressed tRFs/tiRNAs between early LUAD and healthy controls, advanced LUAD and healthy controls, and early LUAD and advanced LUAD. tRFs, TRNA-derived fragments; tiRNAs, tRNA-derived stress-induced RNAs; LUAD, lung adenocarcinoma; PCA, principal component analysis.

As the core standard to measure the rationality and reliability of sample selection, the closer the value of the sample correlation coefficient is to 1, the greater the degree of similarity is between any two samples. In this study, by calculating the sample correlation coefficient, it was confirmed that the selected 12 plasma samples were suitable for this sequencing analysis, and the results were accurate and reliable (**Figure 1A**). The results of the principal component analysis showed significant differences in the expression profiles of tRFs/tiRNAs between LUAD patients and healthy controls (**Figure 1B**). As shown in **Figure 1C**, a total of 506 tRNA derivatives were identified by sequencing analysis of tRFs/tiRNAs, including 431 novel tRNA derivatives that were not annotated in the tRFdb database. Moreover, as shown in **Figure 1D**, there were commonly and specifically expressed tRFs/tiRNAs found between early LUAD patients and healthy controls, advanced LUAD patients and healthy controls, and early and advanced LUAD patients, respectively.

### Analysis of plasma tRF/tiRNA subtypes

**Figure 2:**
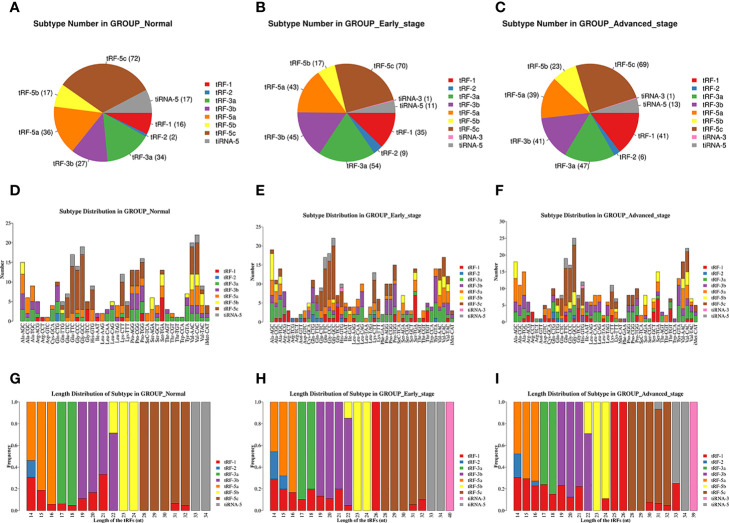
Analysis of plasma tRF/tiRNA subtypes in LUAD patients and healthy controls. **(A–C)** The distribution of tRF/tiRNA subtypes among the healthy controls, early LUAD, and advanced LUAD. **(D–F)** The number of subtypes for tRFs/tiRNAs against tRNA isodecoders among the healthy controls, early LUAD, and advanced LUAD. **(G–I)** The frequency of subtypes against length of tRFs/tiRNAs among the healthy controls, early LUAD, and advanced LUAD. tRFs, TRNA-derived fragments; tiRNAs, tRNA-derived stress-induced RNAs; LUAD, lung adenocarcinoma.

The pie charts of the distribution of tRF/tiRNA subtype revealed that the number of each tRF/tiRNA subtype varied in the early LUAD, advanced LUAD, and healthy control groups (**Figures 2A–C**). The amounts of tRF-1, tRF-3a, tRF-3b, and tRF-5a were increased in early and advanced LUAD patients compared to normal controls. Furthermore, tRF-1 and tRF-5b counts were significantly elevated in advanced LUAD patients compared to early LUAD patients. As can be seen in **Figures 2D–F**, Arg-TCT, Leu-TAA, Phe-GAA, and Ser-CGA contained in LUAD are four tRNA isodecoders present in LUAD patients that were absent in healthy controls. Furthermore, although the tRNA isodecoders had the same anticodon, the types and numbers of subtypes of tRF and tiRNA subtypes were not identical in the healthy control, early LUAD, and advanced LUAD groups. The frequencies of tRF/tiRNA subtypes in tRNAs with different sequence lengths were also not identical, and the present study additionally found that there were also significant differences in the frequencies of tRF/tiRNA subtypes with the same sequence length among the healthy control, early LUAD, and advanced LUAD groups, as indicated in **Figures 2G–I**.

### Differential expression analysis of tRFs/tiRNAs

**Figure 3:**
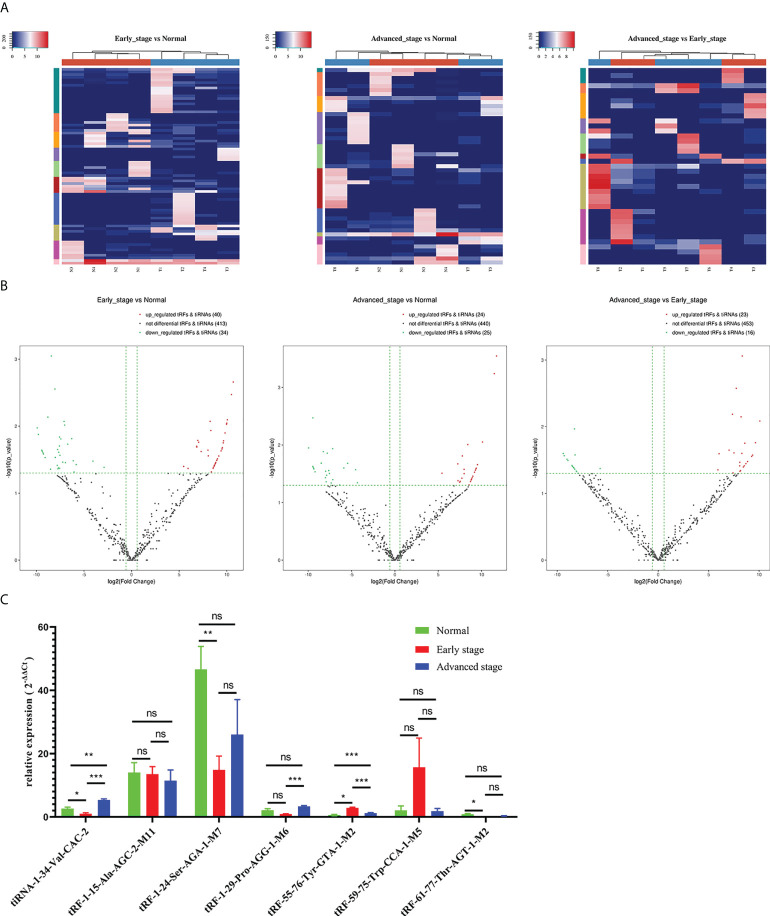
Differential expression analysis of tRFs/tiRNAs in early LUAD, advanced LUAD, and healthy controls. **(A)** The hierarchical clustering heatmap showed the differentially expressed tRFs/tiRNAs between early LUAD and healthy controls, advanced LUAD and healthy controls, and early LUAD and advanced LUAD. **(B)** Volcano plot of differentially expressed tRFs/tiRNAs between early LUAD and healthy controls, advanced LUAD and healthy controls, and early LUAD and advanced LUAD. **(C)** The relative expression levels of seven tRFs/tiRNAs (tiRNA-1:34-Val-CAC-2, tRF-1:15-Ala-AGC-2-M11, tRF-1:24-Ser-AGA-1-M7, tRF-1:29-Pro-AGG-1-M6, tRF-55:76-Tyr-GTA-1-M2, tRF-59:75-Trp-CCA-1-M5, and tRF-61:77-Thr-AGT-1-M2) in healthy controls, early LUAD, and advanced LUAD. *p < 0.05, **p < 0.01, and ***p < 0.001. ns, no significance; tRFs, TRNA-derived fragments; tiRNAs, tRNA-derived stress-induced RNAs; LUAD, lung adenocarcinoma.

The unsupervised hierarchical clustering heatmap demonstrated differential changes in tRF/tiRNA expression between any two groups among the healthy controls group, early LUAD group, and advanced LUAD group (**Figure 3A**). As plotted in **Figure 3B**, 40 upregulated and 34 downregulated tRFs/tiRNAs existed between early LUAD patients and healthy controls; among the differentially expressed tRFs/tiRNAs found between advanced LUAD patients and healthy controls, 24 were upregulated and 25 were downregulated, and there were 23 increased and 16 decreased tRFs/tiRNAs in patients with advanced LUAD compared to early LUAD. Based on the CPM results, seven tRFs/tiRNAs (tiRNA-1:34-Val-CAC-2, tRF-1:15-Ala-AGC-2-M11, tRF-1:24-Ser-AGA-1-M7, tRF-1:29-Pro-AGG-1-M6, tRF-55:76-Tyr-GTA-1-M2, tRF-59:75-Trp-CCA-1-M5, and tRF-61:77-Thr-AGT-1-M2) with good homogeneity and high variability in different groups were selected, and their expression levels were assessed by qRT-PCR. The results showed that the expression levels of tiRNA-1:34-Val-CAC-2, tRF-1:24-Ser-AGA-1-M7, tRF-1:29-Pro-AGG-1-M6, tRF-55:76-Tyr-GTA-1-M2, and tRF-61:77-Thr-AGT-1-M2 varied in LUAD patients (**Figure 3C**). Eventually, according to the relative expression of each tRF/tiRNA, this project chose three tRFs/tiRNAs (tiRNA-1:34-Val-CAC-2, tRF-1:29-Pro-AGG-1-M6, and tRF-55:76-Tyr-GTA-1-M2) with the most significant differential expression as candidate tRFs/tiRNAs for follow-up studies.

### Expression and diagnostic efficacy of candidate plasma tRFs/tiRNAs in lung adenocarcinoma

**Figure 4:**
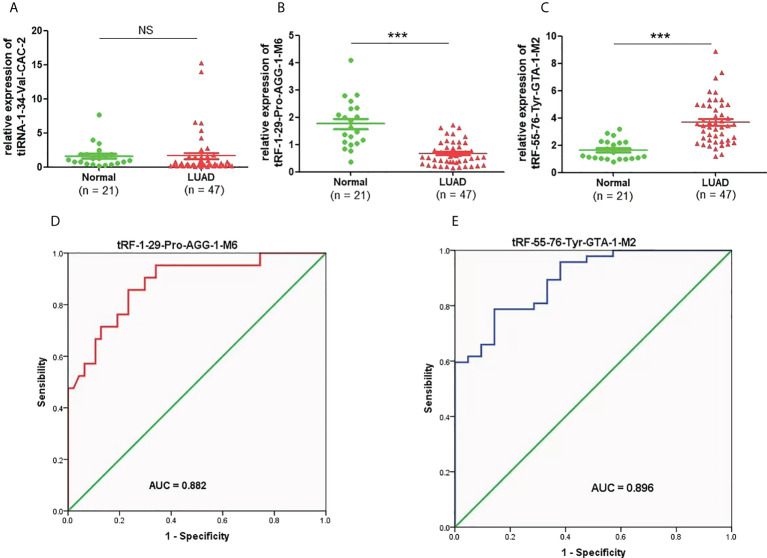
The expression levels and diagnostic value of candidate plasma tRFs/tiRNAs in LUAD. **(A–C)** The relative expression levels of tiRNA-1:34-Val-CAC-2, tRF-1:29-Pro-AGG-1-M6, and tRF-55:76-Tyr-GTA-1-M2 in LUAD patients and normal controls. **(D, E)** The diagnostic performances of tRF-1:29-Pro-AGG-1-M6 and tRF-55:76-Tyr-GTA-1-M2 in LUAD patients ***p < 0.001. ns, no significance. tRFs, TRNA-derived fragments; tiRNAs, tRNA-derived stress-induced RNAs; LUAD, lung adenocarcinoma.

To clarify the concrete expression of tiRNA-1:34-Val-CAC-2, tRF-1:29-Pro-AGG-1-M6, and tRF-55:76-Tyr-GTA-1-M2 in the plasma of LUAD patients, their expression levels were further analyzed by qRT-PCR. Compared to that in healthy controls, the expression level of tRF-1:29-Pro-AGG-1-M6 was significantly downregulated in LUAD patients, while that of tRF-55:76-Tyr-GTA-1-M2 was upregulated distinctly (**Figures 4B, C**). However, as shown in **Figure 4A**, there was no obvious difference in the expression of plasma tiRNA-1:34-Val-CAC-2 in the plasma of LUAD patients compared to that of the healthy controls. On this basis, the diagnostic value of tRF-1:29-Pro-AGG-1-M6 and tRF-55:76-Tyr-GTA-1-M2 in LUAD was further analyzed. The AUCs of tRF-1:29-Pro-AGG-1-M6 and tRF-55:76-Tyr-GTA-1-M2 were 0.882 (95% confidence interval, 0.794–0.970) and 0.896 (95% confidence interval, 0.821–0.970), respectively, and their optimal cutoff values for tRF-1:29-Pro-AGG-1-M6 and tRF-55:76-Tyr-GTA-1-M2 were 0.9575 (sensitivity, 85.7%; specificity, 76.6%) and 2.277 (sensitivity, 78.7%; specificity, 85.7%), respectively (**Figures 4D, E**). It can be concluded that tRF-1:29-Pro-AGG-1-M6 and tRF-55:76-Tyr-GTA-1-M2 have tremendous potential in the diagnosis of LUAD.

### Correlation of tRF-1:29-Pro-AGG-1-M6 and tRF-55:76-Tyr-GTA-1-M2 expression with clinicopathological features

The correlation of the expression levels of tRF-1:29-Pro-AGG-1-M6 and tRF-55:76-Tyr-GTA-1-M2 with clinicopathological features was further evaluated. As shown in **Table 1**, the expression of tRF-1:29-Pro-AGG-1-M6 was related to TNM stage, N stage, and the expression of CEA, while there was no clear correlation with age, gender, T stage, M stage, diameter, or the expression levels of NSE and SCC. Meanwhile, the expression of tRF-55:76-Tyr-GTA-1-M2 in LUAD was significantly correlated with TNM stage, T stage, N stage, M stage, diameter, and the expression of levels CEA and SCC, but not with age, gender, or the expression of NSE.

**Table 1:** The correlation of the expression levels of tRF-1-29-Pro-AGG-1-M6 and tRF-55-76-Tyr-GTA-1-M2 with the clinicopathological characteristics for LUAD patients.

Clinicopathological factor		Cases	tRF-1-29-Pro-AGG-1-M6	*p*-Value	tRF-55-76-Tyr-GTA-1-M2	*p*-Value
Age (years)	<60	32	0.712 ± 0.080	0.318	3.636 ± 0.300	0.721
	≥60	15	0.575 ± 0.099		3.822 ± 0.404	
Gender	Male	19	0.738 ± 0.101	0.368	3.374 ± 0.362	0.274
	Female	28	0.621 ± 0.081		3.913 ± 0.316	
TNM stage	I	29	0.785 ± 0.076	0.017	3.011 ± 0.201	<0.001
	II+III+IV	18	0.480 ± 0.097		4.797 ± 0.426	
T stage	1	31	0.726 ± 0.075	0.208	3.244 ± 0.217	0.007
	≥2	16	0.557 ± 0.111		4.569 ± 0.505	
N stage	0	29	0.785 ± 0.076	0.017	3.011 ± 0.201	<0.001
	≥1	18	0.480 ± 0.097		4.797 ± 0.426	
M stage	0	36	0.734 ± 0.070	0.060	3.190 ± 0.207	<0.001
	≥1	11	0.454 ± 0.124		5.348 ± 0.524	
Diameter	<3	32	0.755 ± 0.072	0.206	3.119 ± 0.207	0.003
	≥3	6	0.509 ± 0.226		4.796 ± 0.508	
	Unknown	9				
CEA	<5 ng/ml	31	0.731 ± 0.067	0.034	3.340 ± 0.223	0.038
	≥5 ng/ml	12	0.448 ± 0.115		4.448 ± 0.609	
	Unknown	4				
NSE	<16.3 ng/ml	31	0.677 ± 0.079	0.510	3.571 ± 0.281	0.286
	≥16.3 ng/ml	10	0.577 ± 0.090		4.189 ± 0.505	
	Unknown	6				
SCC	<2.7 ng/ml	38	0.655 ± 0.067	0.647	3.522 ± 0.249	0.025
	≥2.7 ng/ml	2	0.518 ± 0.109		6.132 ± 1.200	
	Unknown	7				

### Expression and potential value of tRF-1:29-Pro-AGG-1-M6 and tRF-55:76-Tyr-GTA-1-M2 in lung adenocarcinoma preoperatively and postoperatively

**Figure 5:**
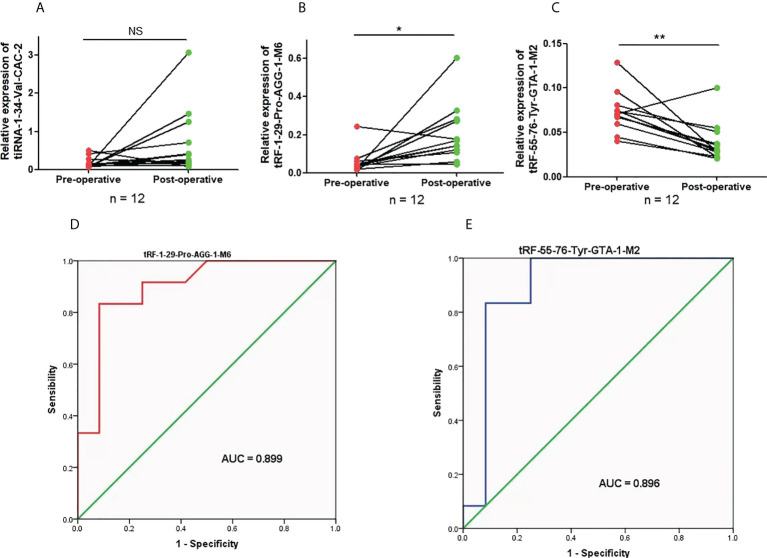
The expression levels and diagnostic value of candidate plasma tRFs/tiRNAs in preoperative and postoperative LUAD. **(A–C)** The relative expression levels of tiRNA-1:34-Val-CAC-2, tRF-1:29-Pro-AGG-1-M6, and tRF-55:76-Tyr-GTA-1-M2 in preoperative and postoperative LUAD patients. **(D, E)** The diagnostic performances of tRF-1:29-Pro-AGG-1-M6 and tRF-55:76-Tyr-GTA-1-M2 in preoperative and postoperative LUAD patients *p < 0.05 and **p < 0.01. ns, no significance. tRFs, TRNA-derived fragments; tiRNAs, tRNA-derived stress-induced RNAs; LUAD, lung adenocarcinoma.

The value of the three candidate tRFs/tiRNAs in monitoring LUAD treatment was evaluated by analyzing their expression in LUAD patients both preoperatively and postoperatively, although there was no divergence in the expression of tiRNA-1:34-Val-CAC-2 after LUAD complete resection (**Figures 5A**). Compared to those before surgery, the expression levels of tRF-1:29-Pro-AGG-1-M6 and tRF-55:76-Tyr-GTA-1-M2 after complete tumor resection were significantly downregulated and upregulated, respectively (**Figures 5B, C**). Further ROC analysis proved that recording the expression levels of tRF-1:29-Pro-AGG-1-M6 and tRF-55:76-Tyr-GTA-1-M2 in LUAD preoperatively and postoperatively can effectively help to distinguish the treatment status of patients, and their AUCs were 0.899 (95% confidence interval, 0.770–1.000) and 0.896 (95% confidence interval, 0.745–1.000), respectively (**Figures 5D, E**). The above findings verify the idea that tRF-1:29-Pro-AGG-1-M6 and tRF-55:76-Tyr-GTA-1-M2 can serve as valuable plasma markers for judging the operative effect in LUAD patients.

### Prediction and functional analysis of potential target genes of tRF-1:29-Pro-AGG-1-M6 and tRF-55:76-Tyr-GTA-1-M2

**Figure 6:**
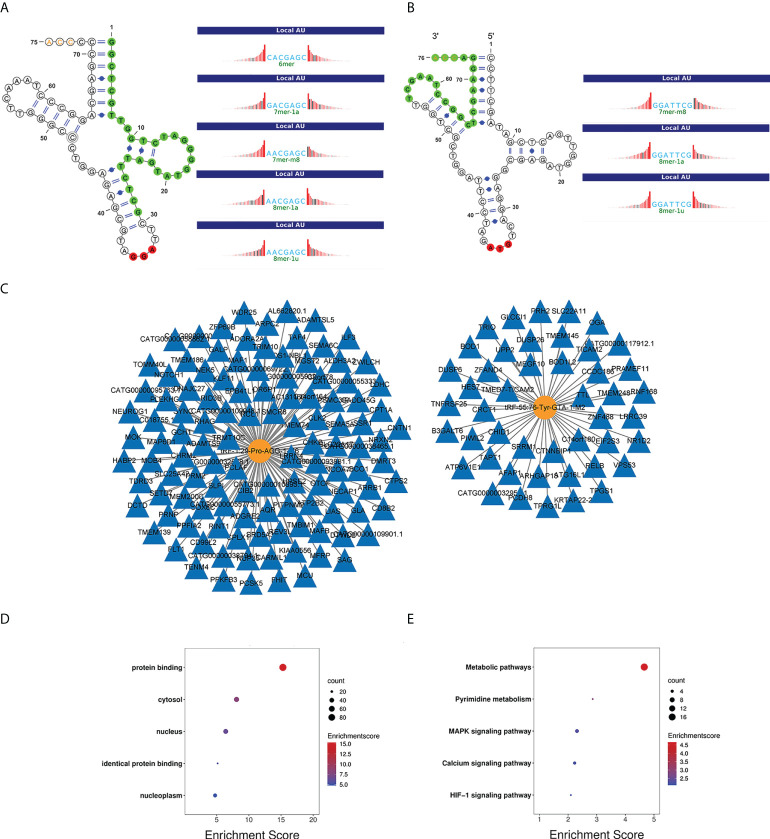
Bioinformatics analysis of tRF-1:29-Pro-AGG-1-M6 and tRF-55:76-Tyr-GTA-1-M2. **(A, B)** Location of the tRF-1:29-Pro-AGG-1-M6 and tRF-55:76-Tyr-GTA-1-M2 in the clover secondary structure of tRNAs and their target sites. **(C)** Target genes of tRF-1:29-Pro-AGG-1-M6 and tRF-55:76-Tyr-GTA-1-M2. **(D)** GO enrichment analysis of tRF-1:29-Pro-AGG-1-M6 and tRF-55:76-Tyr-GTA-1-M2. **(E)** KEGG pathway analysis of tRF-1:29-Pro-AGG-1-M6 and tRF-55:76-Tyr-GTA-1-M2. GO, Gene Ontology; KEGG, Kyoto Encyclopedia of Genes and Genomes.Note. LUAD, lung adenocarcinoma; CEA, carcinoembryonic antigen; NSE, neuron-specific enolase.

In addition to showing the positions of tRF-1:29-Pro-AGG-1-M6 and tRF-55:76-Tyr-GTA-1-M2 on the clover secondary structure of their corresponding tRNAs, **Figures 6A, B** also show their respective target sites. The regulatory network diagrams of tRF-1:29-Pro-AGG-1-M6 and tRF-55:76-Tyr-GTA-1-M2 revealed that one tsRNA corresponded to multiple messenger RNAs (**Figures 6C**). The GO function analysis manifested the finding that the target genes of tRF-1:29-Pro-AGG-1-M6 and tRF-55:76-Tyr-GTA-1-M2 were widely distributed in the cytosol, nucleus, and nucleoplasm and played central roles in cell growth and development by promoting biological processes such as protein binding and identical protein binding (**Figures 6D**). The KEGG pathway enrichment analysis also demonstrated that the target genes of tRF-1:29-Pro-AGG-1-M6 and tRF-55:76-Tyr-GTA-1-M2 were mainly enriched in cancer-related signaling pathways, including the metabolic pathway, pyrimidine metabolism, MAPK signaling pathway, calcium signaling pathway, and HIF-1 signaling pathway (**Figure 6E**).

## Discussion

LUAD is the most common subtype of lung cancer at present. Unfortunately, many patients do not receive timely and effective diagnosis and treatment, leading to a poor prognosis. The emerging biomarker tsRNA has been discussed by more and more researchers owing to its stable existence in the body fluid circulation and is actively used in the diagnosis, treatment, and monitoring of various diseases (7, 21). The latest research suggests that the key members of tsRNAs, tRFs, and tiRNAs are biomarkers with great potential, which can affect the occurrence and development of tumors by acting on protein translation and gene expression in tumor cells (22–24). In this study, the expression profiles of tRFs/tiRNAs in the plasma of LUAD patients were analyzed using high-throughput sequencing technology. It was unexpectedly discovered that 431 new tRFs/tiRNAs were not annotated in the tRFdb database, and a more in-depth study of them in the future will help to unearth their value in LUAD. Furthermore, compared to healthy controls, there were 350 and 344 differentially expressed tRFs/tiRNAs in early LUAD and advanced LUAD patients, respectively. Complementary subtype authentication of tRFs/tiRNAs illuminated the fact that LUAD patients had abnormally elevated levels of tRF-1, tRF-3a, tRF-3b, and tRF-5a. Further analysis also found that the isoforms corresponding to the missing tRNA isodecoders Arg-TCT, Leu-TAA, Phe-GAA, and Ser-CGA in the control group were tRF-1, tRF-3a, tRF-3b, and tRF-5a, which are overexpressed in LUAD. Studies have previously assessed the presence of differentially expressed tRFs/tiRNAs in LUAD tissue samples (25–27). This study revealed the altered tRF/tiRNA expression in the plasma samples of LUAD patients from another perspective, thus also providing support for exploring tRFs/tiRNAs as potential biomarkers for LUAD.

In the present project, seven tRFs/tiRNAs were screened by qRT-PCR, and the results revealed that the expression levels of tiRNA-1:34-Val-CAC-2, tRF-1:29-Pro-AGG-1-M6, and tRF-55:76-Tyr-GTA-1-M2 in LUAD matched with the sequencing consequences. These tRFs/tiRNAs were selected as candidates for further ROC curve analysis, and their further validation of candidate tRFs/tiRNAs demonstrated that, compared to healthy subjects, tRF-55:76-Tyr-GTA-1-M2 was upregulated in LUAD patients, while the expression of tRF-1:29-Pro-AGG-1-M6 was greatly downregulated. In contrast, the AUCs of tRF-1:29-Pro-AGG-1-M6 and tRF-55:76-Tyr-GTA-1-M2 reached 0.882 and 0.896, exhibiting a more obvious diagnostic advantage for LUAD patients. More encouragingly, this study also assessed the correlation between the expression levels of these tRFs/tiRNAs in LUAD and clinicopathological features, and the results revealed that the expression of tRF-55:76-Tyr-GTA-1-M2 was evidently elevated in LUAD patients, indicating that it was positively related to the malignancy of LUAD. Moreover, the expression of tRF-1:29-Pro-AGG-1-M6 was inversely associated with the clinical stage of patients, demonstrating that its high expression has great potential in inhibiting tumor progression. In addition, tRF-1:29-Pro-AGG-1-M6 was downregulated in LUAD patients after complete tumor resection compared to preoperatively, while the expression level of tRF-55:76-Tyr-GTA-1-M2 was reversely upregulated. It can be concluded that the expression levels of tRF-1:29-Pro-AGG-1-M6 and tRF-55:76-Tyr-GTA-1-M2 are closely associated with tumor occupy. Shao et al. (19) found that the upregulated expression of tRF-Leu-CAG in lung cancer is evidently correlated with its progression stage, which is consistent with the results of this experiment. Yang et al. measured plasma AS-tDR-007333 levels and found that they were significantly higher in NSCLC patients than in healthy controls. The study further revealed that the AUC for AS-tDR-007333 was 0.9379, which suggests that AS-tDR-007333 can serve as a diagnostic biomarker for NSCLC (28). Enlightened by previous research carried out by Yang et al., we screened and identified tRF-55:76-Tyr-GTA-1-M2 and tRF-1:29-Pro-AGG-1-M6 as potential biomarkers with high diagnostic sensitivity and specificity in LUAD patients.

Multiple studies have identified arginine (Arg), isoleucine (Leu), phenylalanine (Phe), and serine (Ser) as important components of tumor cell metabolism (29–32), indicating that altered tRF/tiRNA expression profiles could trigger metabolic disturbances in tumors (33). The tRF-Glu-TTC-027 was also found to regulate the progression of gastric carcinoma *via* the MAPK signaling pathway (34). Further exploration of the downstream regulatory mechanism of tRF-55:76-Tyr-GTA-1-M2 and tRF-1:29-Pro-AGG-1-M6 in our research revealed that they participated and functioned in key biological signaling pathways, such as the metabolic pathway, pyrimidine metabolism, calcium signaling pathway, MAPK signaling pathway, and HIF-1 signaling pathway. Our results suggest that tRF-55:76-Tyr-GTA-1-M2 and tRF-1:29-Pro-AGG-1-M6 may affect the occurrence and progression of LUAD through these tumor-related signaling pathways, providing us with a direction to further explore their mechanism.

In conclusion, our study revealed the landscape of tRF/tiRNA expression profiles in plasma with LUAD patients and identified tRF-55:76-Tyr-GTA-1-M2 and tRF-1:29-Pro-AGG-1-M6 as promising, novel biomarkers for the diagnosis of LUAD. However, there are several limitations in the present study that should be pointed out. First, our study included only 47 LUAD patients and 21 healthy controls; thus, the total sample size was small. In addition, the survival analysis data of tRF-55:76-Tyr-GTA-1-M2 and tRF-1:29-Pro-AGG-1-M6 were not analyzed, as we lacked long-term clinical follow-up data. Therefore, further studies with larger sample sizes are required to explore the role and mechanism of these tRFs/tiRNAs in the occurrence and development of LUAD.

## Data availability statement

The original contributions presented in the study are included in the article/supplementary material. Further inquiries can be directed to the corresponding authors.

## Ethics statement

This study was approved by the responsible committee for human experimentation of Fujian Provincial Hospital (K2021-03-054). The patients/participants provided their written informed consent to participate in this study.

## Author contributions

JY, XX, and LC designed and directed this study. GY, YW, and SH performed the experiments. QC and CH were responsible for collecting patients’ serum samples and clinical information. JY, XL, and LC performed the statistical analysis. JY and GY wrote the manuscript. FC, XX, and LC reviewed and edited the manuscript. All authors contributed to the article and approved the submitted version.

## Funding

This work was supported by the Fujian Natural Science Foundation of China (No. 2021J1375, No. 2020J05259) and Joint Funds for the Innovation of Science and Technology of Fujian Province (No. 2019Y9025) Startup Fund for Scientific Research of Fujian Medical University (No.2021QH1316).

## Conflict of interest

The authors declare that the research was conducted in the absence of any commercial or financial relationships that could be construed as a potential conflict of interest.

## Publisher’s note

All claims expressed in this article are solely those of the authors and do not necessarily represent those of their affiliated organizations, or those of the publisher, the editors and the reviewers. Any product that may be evaluated in this article, or claim that may be made by its manufacturer, is not guaranteed or endorsed by the publisher.

## References

[1] Sung H Ferlay J Siegel RL Laversanne M Soerjomataram I Jemal A. Global cancer statistics 2020: GLOBOCAN estimates of incidence and mortality worldwide for 36 cancers in 185 countries. CA: Cancer J Clin (2021) 71(3):209–49. doi: 10.3322/caac.21660 33538338

[2] Zhang H Guo L Chen J. Rationale for lung adenocarcinoma prevention and drug development based on molecular biology during carcinogenesis. OncoTargets Ther (2020) 13:3085–91. doi: 10.2147/OTT.S248436 PMC7166063 32341654

[3] Al-Dherasi A Liao Y Al-Mosaib S Hua R Wang Y Yu Y. Allele frequency deviation (AFD) as a new prognostic model to predict overall survival in lung adenocarcinoma (LUAD). Cancer Cell Int (2021) 21(1):451. doi: 10.1186/s12935-021-02127-z 34446004 PMC8390239

[4] National Lung Screening Trial Research Team Aberle,DR Adams AM Berg CD Black WC Clapp JD. Reduced lung-cancer mortality with low-dose computed tomographic screening. New Engl J Med (2011) 365(5):395–409. doi: 10.1056/NEJMoa1102873 21714641 PMC4356534

[5] I,H Cho,JY. Lung cancer biomarkers. Adv Clin Chem (2015) 72:107–70. doi: 10.1016/bs.acc.2015.07.003 26471082

[6] Zhou L Li X Liu Q Zhao F Wu J. Small RNA transcriptome investigation based on next-generation sequencing technology. journal of genetics and genomics. Yi Chuan xue bao (2011) 38(11):505–13. doi: 10.1016/j.jgg.2011.08.006 22133681

[7] Xu WL Yang Y Wang YD Qu LH Zheng,LL. Computational approaches to tRNA-derived small RNAs. Non-coding RNA (2017) 3(1):2. doi: 10.3390/ncrna3010002 PMC5832003 29657274

[8] Saikia M Hatzoglou M. The many virtues of tRNA-derived stress-induced RNAs (tiRNAs): Discovering novel mechanisms of stress response and effect on human health. J Biol Chem (2015) 290(50):29761–8. doi: 10.1074/jbc.R115.694661 PMC4705977 26463210

[9] Borek E Baliga BS Gehrke CW Kuo CW Belman S Troll W. High turnover rate of transfer RNA in tumor tissue. Cancer Res (1977) 37(9):3362–6. 884680

[10] Honda S Loher P Shigematsu M Palazzo JP Suzuki R Imoto I. Sex hormone-dependent tRNA halves enhance cell proliferation in breast and prostate cancers. Proc Natl Acad Sci United States America (2015) 112(29):E3816–25. doi: 10.1073/pnas.1510077112 PMC4517238 26124144

[11] Sharma U Conine CC Shea JM Boskovic A Derr AG Bing XY. Biogenesis and function of tRNA fragments during sperm maturation and fertilization in mammals. Sci (New York N.Y.) (2016) 351(6271):391–6. doi: 10.1126/science.aad6780 PMC4888079 26721685

[12] Godoy PM Bhakta NR Barczak AJ Cakmak H Fisher S MacKenzie TC. Large Differences in small RNA composition between human biofluids. Cell Rep (2018) 25(5):1346–58. doi: 10.1016/j.celrep.2018.10.014 PMC6261476 30380423

[13] Kuscu C Kumar P Kiran M Su Z Malik A Dutta A. tRNA fragments (tRFs) guide ago to regulate gene expression post-transcriptionally in a dicer-independent manner. RNA (New York N.Y.) (2018) 24(8):1093–105. doi: 10.1261/rna.066126.118 PMC6049499 29844106

[14] Lyons SM Kharel P Akiyama Y Ojha S Dave D Tsvetkov V. eIF4G has intrinsic G-quadruplex binding activity that is required for tiRNA function. Nucleic Acids Res (2020) 48(11):6223–33. doi: 10.1093/nar/gkaa336 PMC7293036 32374873

[15] Watanabe T Tomizawa S Mitsuya K Totoki Y Yamamoto Y Kuramochi-Miyagawa S. Role for piRNAs and noncoding RNA in *de novo* DNA methylation of the imprinted mouse Rasgrf1 locus. Sci (New York N.Y.) (2011) 332(6031):848–52. doi: 10.1126/science.1203919 PMC3368507 21566194

[16] Oberbauer V Schaefer,MR. tRNA-derived small RNAs: Biogenesis, modification, function and potential impact on human disease development. Genes (2018) 9(12):607. doi: 10.3390/genes9120607 PMC6315542 30563140

[17] Chen B Liu S Wang H Li G Lu X Xu H. Differential expression profiles and function prediction of transfer RNA-derived fragments in high-grade serous ovarian cancer. BioMed Res Int (2021) 2021:5594081. doi: 10.1155/2021/5594081 33860037 PMC8028742

[18] Papadimitriou MA Avgeris M Levis P Papasotiriou EC Kotronopoulos G Stravodimos K. tRNA-derived fragments (tRFs) in bladder cancer: Increased 5’-tRF-LysCTT results in disease early progression and patients’ poor treatment outcome. Cancers (2020) 12(12):3661. doi: 10.3390/cancers12123661 PMC7762106 33291319

[19] Shao Y Sun Q Liu X Wang P Wu R Ma Z. tRF-Leu-CAG promotes cell proliferation and cell cycle in non-small cell lung cancer. Chem Biol Drug design (2017) 90(5):730–8. doi: 10.1111/cbdd.12994 PMC5697697 28378898

[20] Li J Cao C Fang L Yu W. Serum transfer RNA-derived fragment tRF-31-79MP9P9NH57SD acts as a novel diagnostic biomarker for non-small cell lung cancer. J Clin Lab Anal (2022) 36(7). doi: 10.1002/jcla.24492 PMC9279995 35576497

[21] Zhang Y Bi Z Dong X Yu M Wang K Song X. tRNA-derived fragments: tRF-Gly-CCC-046, tRF-Tyr-GTA-010 and tRF-Pro-TGG-001 as novel diagnostic biomarkers for breast cancer. Thorac Cancer (2021) 12(17):2314–23. doi: 10.1111/1759-7714.14072 PMC8410570 34254739

[22] Ivanov P Emara MM Villen J Gygi SP Anderson P. Angiogenin-induced tRNA fragments inhibit translation initiation. Mol Cell (2011) 43(4):613–23. doi: 10.1016/j.molcel.2011.06.022 PMC3160621 21855800

[23] Haussecker D Huang Y Lau A Parameswaran P Fire AZ Kay,MA. Human tRNA-derived small RNAs in the global regulation of RNA silencing. RNA (New York N.Y.) (2010) 16(4):673–95. doi: 10.1261/rna.2000810 PMC2844617 20181738

[24] Zhu P Yu J Zhou P. Role of tRNA-derived fragments in cancer: novel diagnostic and therapeutic targets tRFs in cancer. Am J Cancer Res (2020) 10(2):393–402. 32195016 PMC7061753

[25] Zhang J Li L Luo L Yang X Zhang J Xie Y. Screening and potential role of tRFs and tiRNAs derived from tRNAs in the carcinogenesis and development of lung adenocarcinoma. Oncol Lett (2021) 22(1):506. doi: 10.3892/ol.2021.12767 33986867 PMC8114470

[26] Wang J Ma G Li M Han X Xu J Liang M. Plasma tRNA fragments derived from 5’ ends as novel diagnostic biomarkers for early-stage breast cancer. Mol Ther Nucleic Acids (2020) 21:954–64. doi: 10.1016/j.omtn.2020.07.026 PMC7452045 32814252

[27] Huang LT Cui M Silva M Okuda K Shimada Y Wang JH. Expression profiles of tRNA-derived fragments and their potential roles in lung adenocarcinoma. Ann Trans Med (2022) 10(4):196. doi: 10.21037/atm-22-119 PMC8908152 35280355

[28] Yang W Gao K Qian Y Huang Y Xiang Q Chen C. A novel tRNA-derived fragment AS-tDR-007333 promotes the malignancy of NSCLC *via* the HSPB1/MED29 and ELK4/MED29 axes. J Hematol Oncol (2022) 15(1):53. doi: 10.1186/s13045-022-01270-y 35526007 PMC9077895

[29] Kumari N Bansal S. Arginine depriving enzymes: applications as emerging therapeutics in cancer treatment. Cancer chemother Pharmacol (2021) 88(4):565–94. doi: 10.1007/s00280-021-04335-w 34309734

[30] Sivanand S Vander Heiden,MG. Emerging roles for branched-chain amino acid metabolism in cancer. Cancer Cell (2020) 37(2):147–56. doi: 10.1016/j.ccell.2019.12.011 PMC7082774 32049045

[31] Liang KH Cheng ML Lo CJ Lin YH Lai MW Lin WR. Plasma phenylalanine and glutamine concentrations correlate with subsequent hepatocellular carcinoma occurrence in liver cirrhosis patients: an exploratory study. Sci Rep (2020) 10(1):10926. doi: 10.1038/s41598-020-67971-x 32616821 PMC7331577

[32] Kinslow CJ Sun RC Chaudhary KR Cheng,SK. “Serine and one-carbon metabolism in breast cancer metastasis”-letter. Mol Cancer Res (2020) 18(11):1755. doi: 10.1158/1541-7786 33144383 PMC8588402

[33] Chen Q Yan M Cao Z Li X Zhang Y Shi J. Sperm tsRNAs contribute to intergenerational inheritance of an acquired metabolic disorder. Sci (New York N.Y.) (2016) 351(6271):397–400. doi: 10.1126/science.aad7977 26721680

[34] Xu W Zhou B Wang J Tang L Hu Q Wang J. tRNA-derived fragment tRF-Glu-TTC-027 regulates the progression of gastric carcinoma *via* MAPK signaling pathway. Front Oncol (2021) 11:733763. doi: 10.3389/fonc.2021.733763 34497772 PMC8419445

